# Predictors of Peripheral Retinal Non-Perfusion in Clinically Significant Diabetic Macular Edema

**DOI:** 10.3390/jcm14010052

**Published:** 2024-12-26

**Authors:** Martin Hein, Andrew Mehnert, Fiona Josephine, Arman Athwal, Dao-Yi Yu, Chandrakumar Balaratnasingam

**Affiliations:** 1 Lions Eye Institute, Perth, WA 6009, Australia; 2 Centre for Ophthalmology and Visual Science, University of Western Australia, Perth, WA 6009, Australia; 3 School of Engineering Science, Simon Fraser University, Burnaby, BC V5A 1S6, Canada; 4 Department of Medical Physics and Biomedical Engineering, University College London, London WC1E 6BT, UK; 5 Department of Ophthalmology, Sir Charles Gairdner Hospital, Perth, WA 6009, Australia

**Keywords:** diabetic macular edema, peripheral non-perfusion, retinal ischemia, OCTA, vessel density

## Abstract

**Background/Objectives:** Diabetic macular edema (DME) is a significant cause of vision loss. The development of peripheral non-perfusion (PNP) might be associated with the natural course, severity, and treatment of DME. The present study seeks to understand the predictive power of central macular changes and clinico-demographic features for PNP in patients with clinically significant DME. **Methods**: A prospective study using contemporaneous multi-modal retinal imaging was performed. In total, 48 eyes with DME from 33 patients were enrolled. Demographic, clinical history, laboratory measures, ultrawide field photography, fluorescein angiography, optical coherence tomography (OCT), and OCT angiography results were acquired. Anatomic and vascular features of the central macula and peripheral retina were quantified from retinal images. Separate (generalized) linear mixed models were used to assess differences between PNP present and absent groups. Mixed effects logistic regression was used to assess which features have predictive power for PNP. **Results**: Variables with significant differences between eyes with and without PNP were insulin use (*p* = 0.0001), PRP treatment (*p* = 0.0003), and diffuse fluorescein leakage (*p* = 0.013). Importantly, there were no significant differences for any of the macular vascular metrics including vessel density (*p* = 0.15) and foveal avascular zone (FAZ) area (*p* = 0.58 and capillary tortuosity (*p* = 0.55). Features with significant predictive power (all *p* < 0.001) were subretinal fluid, FAZ eccentricity, ellipsoid zone disruption, past anti-VEGF therapy, insulin use, and no ischemic heart disease. **Conclusions**: In the setting of DME, macular vascular changes did not predict the presence of PNP. Therefore, in order to detect peripheral non-perfusion in DME, our results implicate the importance of peripheral retinal vascular imaging.

## 1. Introduction

Diabetic macular edema (DME) is one of the most common causes of vision loss in patients with diabetes [1]. The pathogenic mechanisms underlying DME are complex, and the breakdown of the blood–retina barrier can be secondary to a host of cytokine and chemokine upregulation within the macular milieu [2]. Significant advancements in structural optical coherence tomography (OCT), OCT angiography (OCTA), and ultrawide field imaging (UWF) technology have significantly improved our understanding of vascular endothelial growth factor (VEGF)- and inflammatory-mediated pathways in DME development [3]. This knowledge has been used in clinical practice to help choose the most appropriate treatment for DME (intravitreal anti-VEGF, corticosteroids, and laser). Multimodal imaging biomarkers of the macula are also useful for stratifying patients that are at risk of developing DME, identifying patients that may have a sub-optimal response to anti-VEGF therapy and predicting long-term visual outcomes [4,5].

Peripheral retinal non-perfusion (PNP) is another complication of diabetes that can lead to vision loss through retinal neovascularization, vitreous hemorrhage, and tractional retinal detachment [6]. Several lines of evidence suggest that the pathogenesis of PNP, similar to DME, is multifactorial [7], such as the fact that (1) the topologic patterns of PNP are not homogeneous, and distinct patterns of retinal PNP have been described in large cohort studies of diabetes [6,8,9] and (2) the progression of PNP can occur despite antagonizing the effects of VEGF with frequent intravitreal therapy [10,11,12]. The peripheral retina is significantly more challenging to visualize than the macula, and image artifacts are more commonly associated with peripheral retinal imaging due to pupil size and aberrations induced by the curvature of the eye. DME is not uncommonly associated with PNP, suggesting that the two manifestations may share common pathogenic links [11,13,14]. Exploring relationships between DME and PNP may improve our understanding of the pathogenesis of diabetic retina disease and possibly unravel novel ways to detect PNP using biomarkers of DME.

In this report, we perform contemporaneous, state-of-the-art retina imaging to investigate associations between DME and retinal PNP. We seek to determine if multimodal imaging features of DME as seen with high-resolution OCT, OCTA, fluorescein angiography (FA), and color photography can predict the presence of PNP. The purpose of this report is to refine pathogenic links between DME and PNP and thereby improve the management of diabetic retina-disease.

## 2. Materials and Methods

### 2.1. Subjects

Participants were recruited from Lions Eye Institute and Sir Charles Gairdner Hospital in Perth, Western Australia. Eligible patients were at least 18 years of age with type 1 or 2 diabetes mellitus (DM) and moderate-to-severe NPDR or PDR with the presence of DME assessed to be the cause of vision loss. DME was determined by OCT features and central subfield thickness (CST) ≥ 250µm. Exclusion criteria were as follows: (1) any coexistent disease that causes retinal ischemia such as retinal artery or vein occlusion; (2) previous intravitreal corticosteroids or anti-VEGF therapy or laser photocoagulation therapy (central or pan-retinal photocoagulation; PRP) within 6 months of the study visit; (3) intraocular pressure ≥ 21 mmHg; (4) low-quality retinal imaging that prohibited qualitative or quantitative analysis; and (5) uncontrolled hypertension. A patient could have both eyes included if eligible.

### 2.2. Retinal Imaging

A standardized protocol for retinal imaging was followed and included the following:

**Color fundus photography:** Retinal photographs of the posterior pole were attained with a Canon CX-1 digital retinal camera (Retinal Imaging Control Software for CX-1, 4.6.0.5, Canon Medical Systems, Otawara, Japan). UWF retinal photographs of the posterior pole and periphery were captured using Optos 200Tx (Optos, Dunfermline, Scotland, UK).

**Structural optical coherence tomography:** OCT images were captured using a Heidelberg Spectralis OCT2 machine (Heidelberg Engineering, Heidelberg, Germany). A raster scan protocol centered on the fovea (scan field 30° × 25°) containing 61 B-scans (122 µm interscan distance) with ART mode enabled (9 frames averaged per B-scan) was used.

**30° and ultrawide field fluorescein angiography:** Several frames of the first 5-min angiogram sequence were taken using the Heidelberg Spectralis OCT2 device (Heidelberg Eye Explorer, 1.10.4.0, Heidelberg Engineering, Heidelberg, Germany). A 30-degree scan angle with ART mode enabled (22 images averaged) was used. Multiple frames of UWF FA images, to visualize the retinal periphery, were then attained for the timeframe 5–10 min after fluorescein administration using Optos 200Tx (Optos, Dunfermline, Scotland, UK).

**Optical coherence tomography angiography:** OCTA images centered on the fovea were captured using the Optovue XR Avanti (RTVue XR. 2018.1.0.43, Optovue, Inc., Freemont, CA, USA) and/or the Heidelberg Spectralis OCT2 Module (Heidelberg Eye Explorer, 1.10.4.0, Heidelberg Engineering, Heidelberg, Germany).

The Optovue XR Avanti has an A-scan rate of 70,000 scans per second, using a light source centered on 840 nm and a bandwidth of 45 nm. It generates OCTA images using the split spectrum amplitude decorrelation angiography (SSADA) algorithm. Two strategies are employed to correct for motion artifacts (originating from blinking, fixation drifts, and micro-saccades). First, it performs real-time eye tracking during acquisition to detect motion and reacquires portions of the corrupted data. Second, it acquires two OCTA volumes orthogonal in the scan direction to one another and combines the information from each during post-processing to obtain the best spatial registration of A-scans. The system automatically segments (delineates) the retinal layers and produces a 2D OCTA *en face* visualization (projection) through selected layers.

For this study, *en face* OCTA images were acquired using a scan area of 3 × 3 mm centered on the fovea through the full retinal slab (scan density of 304). Each image was acquired in approximately 3 s. Images containing significant residual motion artifacts (including ghosting of vessels, streaks, and shear) were excluded from analysis. Only OCTA images with a signal strength index above 50 or a scan quality score above 8 out of 10 were included in this study.

The Heidelberg Spectralis OCT2 device has an A-scan rate of 85,000 scans per second, using a light source centered on 870 nm and a bandwidth of 50 nm. It generates OCTA images using a full-spectrum probabilistic approach that incorporates both phase and amplitude information. It uses a second laser beam to actively track the fundus during acquisition to minimize motion artifacts. The system automatically segments the retinal layers and produces a 2D OCTA *en face* visualization through selected layers. In this study, 3.1 × 3.1 mm *en face* OCTA images (10° × 10°) centered at the fovea were acquired (scan density of 512) through all retinal layers, with ART mode enabled (four frames averaged per B-scan) and projection artifact removal (PAR) enabled.

**Axial length measurement and keratometry:** Axial length and keratometry measurements were recorded using the IOL Master 700 (Carl Zeiss Meditec AG, Jena, Germany). These quantifications, in combination with refraction, were used to correct for image magnification errors on OCTA scans for an accurate measurement of foveal metrics such as the FAZ area.

### 2.3. Quantitative Metrics Derived from OCTA

**Figure 1:**
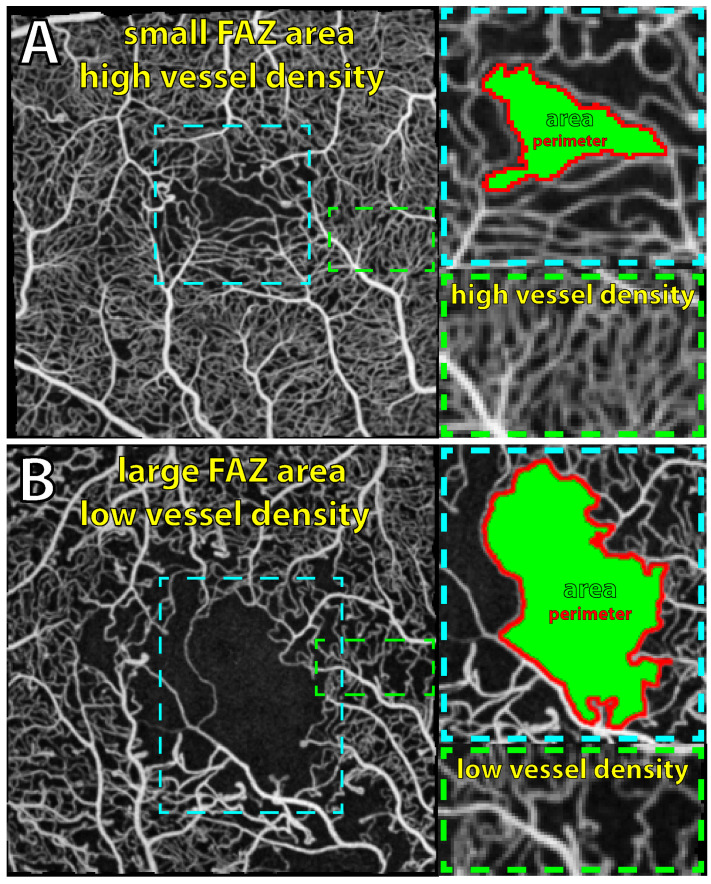
Quantitative macular vascular metrics using optical coherence tomography angiography (OCTA) in eyes with diabetic macula edema. Normal (**A**) and decreased vessel density (**B**) (diabetic macular ischemia) are seen with enlargement of the foveal avascular zone area (FAZ) and FAZ perimeter (cyan insets). The magnified image of macular capillaries (green insets) shows a marked decrease in capillary vessel density in (**B**). OCTA scans are full-retinal-thickness projections captured with Optovue XR Avanti using a 3 × 3 mm scan area centered on the fovea. Images are max intensity projections of 8–10 consecutive OCTA scans from the same eye.

We captured 8–10 consecutive high-quality *en face* OCTA images through the full retinal slab within a period of 5 min from the study eye using the Optovue system for quantitative analysis. Multiple OCTA images taken consecutively allow for image averaging to improve the signal-to-noise ratio, as described in our previous publications [15]. OCTA images from the Heidelberg system were used alongside Optovue OCTA images for qualitative assessment by graders. We did not stratify the macular plexus into superficial and deep vascular plexuses due to edema-associated distortions in the retinal layers causing imprecise layer segmentation. The *en face* OCTA images and corresponding *en face* OCT images were exported and processed using in-house image analysis scripts written in R, FIJI, and MATLAB [16,17,18]. We have previously described the processing steps in detail in our previous publications. Briefly, the *en face* OCTA images were corrected for intensity inhomogeneity, spatially aligned (sequence of translation alignment, affine registration, and elastic alignment), intensity normalized, averaged to create a single image with an improved signal-to-noise ratio, and the single-pixel-thick vessel centerlines were segmented. In addition, following our previous approach, we defined vessels of Horton–Strahler order 2 to 4 to be arterioles and venules and interactively segmented (delineated) these in FIJI [19]. The following features were then computed:

**Macula vessel density (Figure 1):** The microvasculature in the average *en face* OCTA image was segmented (binarized) using a convolutional neural network (CNN), as previously described [20]. Perfusion density (PD) was calculated as the ratio of vessel pixels to total pixels, excluding those pixels belonging to the previously segmented arterioles and venules. Vessel density (VD) was similarly calculated but using a skeletonized version of the binary image, which ignores vessel width.

**Figure 2:**
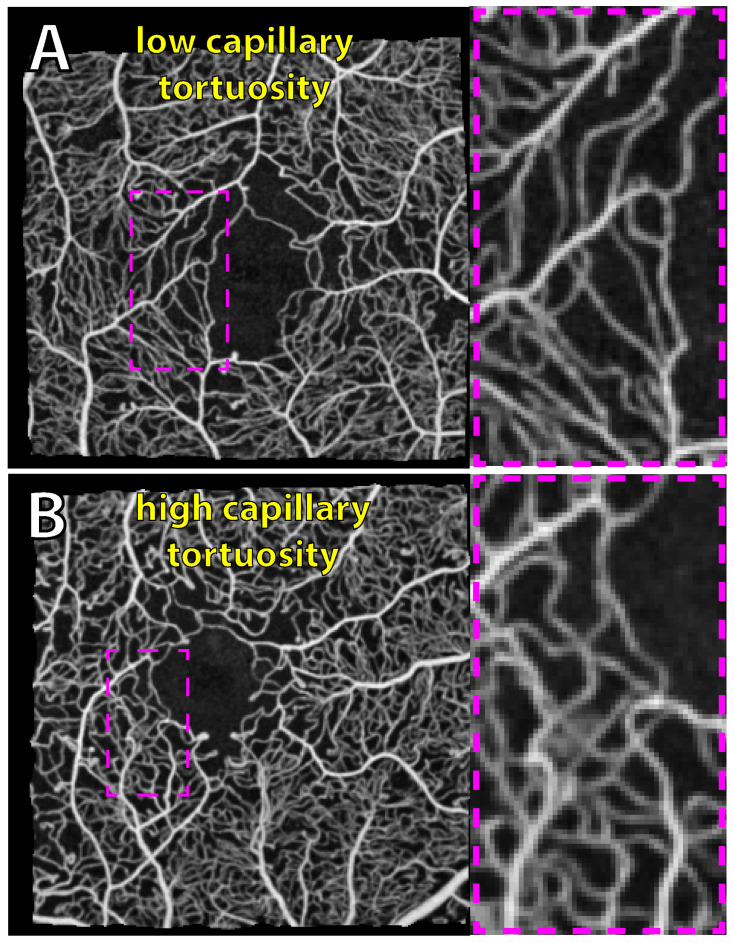
Capillary tortuosity variance between two eyes with diabetic macular edema. Optical coherence tomography angiography (OCTA) scans using a 3 × 3 mm scan area centered on the fovea are used to generate vessel tortuosity metrics. Examples of capillary tortuosity variability are shown in low-tortuosity (**A**) and high-tortuosity (**B**) examples of the central macula; this quantification excludes arterioles and venules. Magnified insets of the temporal terminal capillaries are shown (magenta insets). OCTA scans are full-retinal-thickness projections captured with the Optovue XR Avanti device. Images are max intensity projections of 8–10 consecutive OCTA scans from the same eye.

**Macula vessel tortuosity (Figure 2):** Using the vessel centerline segmentation together with the segmentation of arterioles and venules, two tortuosity measures were calculated for each macula arteriole, venule, and capillary. The first measure (tortuosity method 1) is simply the path length between the vessel endpoints divided by the Euclidean distance between them. The second measure (tortuosity method 2) is the method proposed by Khansari et al. (2017) [21], based on a combination of local and global centerline features.

**Foveal avascular zone metrics (Figure 1):** The following FAZ features were calculated from the average *en face* OCTA image: total area (mm^2^), eccentricity (eccentricity of the ellipse that has the same second moments), the axis ratio (ratio of the height to width of the bounding box of the FAZ), perimeter (mm), and the acircularity index (normalized ratio of area to squared perimeter). Image magnification errors were corrected for using the individual eye’s axial length, keratometry, refraction, and the Littman and modified Bennett formula [22].

### 2.4. Reviewer Grading of Retinal Images

Retinal images were assessed individually by two qualified retinal specialists in a blinded fashion. A third retinal specialist resolved grading disagreements. Multimodal imaging was utilized to grade the following features according to definitions from our previous publication [5]:

**Presence and number of microaneurysms in the macula** [5]: Color photography, OCT, and FA scans were used to define the microaneurysm count. Multiple imaging modalities allowed for the differentiation of microaneurysms from retinal hemorrhages. In the 5.5 mm diameter macula area, microaneurysms were counted as being absent, less than 10 microaneurysms, or 10 or more microaneurysms in number.

**Presence or absence of exudate in the macula** [5]: Color photography and OCT scans were utilized to define exudation and distinguish from hyperreflective foci (HRF). Exudation in the macula area was graded as being present or absent.

**Pattern of fluorescein leakage in the macula** [5]: Leakage patterns on FA frames in the macula were defined using grades from the ETDRS into focal, intermediate, or diffuse leaks [23]. Focal leak was categorized as fluorescein leakage predominantly (>67%) from microaneurysms. Diffuse leak was leakage predominantly from dilated capillaries (<33% from microaneurysms). Intermediate leak was leakage occurring equally from microaneurysms and dilated capillaries.

**Presence/absence of peripheral non-perfusion** [5]: An ETDRS 7-field grid overlay on a UWF FA frame was used to grade the presence or absence of PNP outside the ETDRS grid. Images were evaluated in a binary manner, present or absent, for the occurrence of any degree of PNP.

**Structural OCT volumes of the macula** were evaluated for the following features [5]: presence/absence of subretinal fluid; presence/absence of cysts in the inner nuclear layer (INL); presence/absence of cysts in the outer nuclear layer (ONL); and presence of disorganization of retinal inner layers (DRiL). DRiL was defined as areas for which any boundaries between the ganglion cell–inner plexiform layer complex, INL, and ONL could not be identified, as well as disruption of the external limiting membrane (ELM) and ellipsoid zone (EZ). The central 5.5 mm of the macula was evaluated. Disruption of the EZ and ELM was categorically graded as being present or absent. A “present” grading was denoted if any degree of disruption to the EZ or ELM was evident, or the presence/absence of intraretinal HRF. OCT-derived measurements of retinal thickness were used. The 1 mm, 3 mm, and 6 mm CRT were recorded from the macular thickness ETDRS.

**OCTA images** from both Optovue and Heidelberg devices, including all vascular layers, were used for analysis [5]. Images were graded for the following features: perifoveal capillary loss, categorically graded as being present or absent, and the integrity of the terminal foveal capillary ring, categorically graded as being intact or disrupted.

### 2.5. Statistical Analysis

Statistical analyses were performed in R using a 5% level of significance unless stated otherwise. The final dataset of 55 features/variables (29 numeric and 26 categorical) is summarized in Supplementary Table S1. They include Patient-ID and grouping variable PNP present (Y/N). Several eyes were excluded from the study to maximize the number of patients included whilst at the same time minimizing the number of missing observations (no more than two missing observations—see Results). Missing values were imputed using the method of Stekhoven et al. [24] (non-parametric approach based on random forests suitable for mixed-type data) implemented in the *missForest* R package (v1.5) [25].

The data were summarized graphically for the grouping PNP present using side-by-side boxplots for numeric variables and side-by-side bar plots for categorical variables.

**Univariate analysis of the difference between each group**: As an exploratory first step, a hypothesis test was performed for each variable in turn to determine whether there was a statistically significant difference between the two groups. For the numeric variables, an independent sample *t*-test was performed. For the categorical variables, Fisher’s exact test was performed. For the cases where the result was statistically significant at α = 10%, the variable was tested again, this time using a (generalized) linear mixed model to account for possible within-subject correlation given the data contained both eyes for 15 patients. It was decided that the FAZ area variable and both capillary tortuosity variables would be tested again using separate linear mixed models (whether or not initially statistically significant at α = 10%) because there is a strong physiologic basis and previous reports suggest significant differences [26,27,28,29,30,31,32,33]. In each case, the mixed model was fitted using the measurement as the response variable, the grouping variable as the fixed effect, and Patient-ID as the random effect. For the numeric variables, a linear mixed model was fitted using the *lme4* R package (v1.1-35.5) [34], and the Shapiro–Wilk test and Levene tests were used to assess whether the model assumptions of normality of the residuals and homogeneity of variance were satisfied. For the categorical variables, *lme4* was used to fit a logistic mixed model for binary outcomes and the package *gamlss* (v5.4-22) [35] was used to fit a multinomial mixed model for more than two outcomes.

**Multivariate mixed effects analysis:** The significant variables from the univariate tests do not necessarily translate to significant predictors in a multivariate model [36]. For this reason, mixed effects logistic regression was performed with stepwise variable selection to determine the most discriminatory subset of variables. Specifically, PNP present was taken to be the response variable and Patient-ID as the random effect, and the *buildmer* R package (v2.11) [37] was used to perform backward elimination of variables using the AIC criterion. The classification accuracy of the selected model was assessed in terms of the receiver operating characteristic curve (ROC) using leave-one-out cross validation. The package *pROC* (v1.18.5) [38] was used to estimate the area under the curve (AUC) and associated 95% confidence interval.

## 3. Results

### 3.1. Subjects and Demographics

The final dataset consisted of measurements/observations of 53 variables on 48 eyes from 33 patients. Missing observations were imputed as described in the Statistical Analysis section, with one for “HbA1c” and two for each of the variables “Duration of DM”, “LDL”, “HDL”, “past intravitreal anti-VEGF”, and “Past intravitreal steroids”. The observations included 26 eyes without the presence of PNP and 22 eyes with the presence of PNP. Graphical summaries of the numerical and categorical observations grouped by the presence/absence of PNP are shown in Supplementary Figures S1 and S2. A tabulated summary of observations grouped by the presence/absence of PNP is shown in Table 1, Table 2 and Table 3.

### 3.2. Predictors of Peripheral Non-Perfusion in DME

**Univariate analysis:** The *p*-values for the univariate tests are shown in Table 1, Table 2 and Table 3. The following variables demonstrated a statistically significant difference (*t*-test or Fisher’s exact test) at α = 10% between Y and N of the grouping variable PNP present: insulin, PRP, microaneurysms, pattern of fluorescein leakage, presence of subretinal fluid, occurrence of intraretinal cystoid changes in the ONL, age, perfusion density, and vessel density. FAZ area and both capillary tortuosity measures were included as well. Separate mixed models were fitted with each variable as the response variable, respectively. It was necessary to transform age by raising it to a power of 1.05 and to transform FAZ area by taking the square root to satisfy model assumptions. It was not possible to fit models with microaneurysms, occurrence of intraretinal cystoid changes in the ONL, or capillary tortuosity method 2 as response variables (singular model fits). From the fitted models, only insulin, PRP, and pattern of fluorescein leakage demonstrated a statistically significant difference between Y and N at α = 10% and indeed at the α = 5% level of significance.

**Multivariate analysis (best subset selection):** Given the small number of observations (48 eyes) relative to the number of features/variables (53), it was not possible to fit the full mixed effects logistic regression model. So, as a first step, given the expected high correlation between the two tortuosity measures and also between perfusion density and vessel density, a paired plot was constructed, as shown in Supplementary Figure S3. It was decided to exclude the variable of venules tortuosity method 2 from variable selection because it was strongly correlated with arterioles tortuosity method 2 (*r* = 0.929) and moderately with both arterioles tortuosity method 1 and venules tortuosity method 1. Similarly, it was decided to exclude arterioles tortuosity method 1 because it was strongly correlated with venules tortuosity method 1 (*r* = 0.999) and moderately with venules tortuosity method 2. PRP was also excluded, as PNP is itself an indication for PRP treatment. The backward elimination strategy used in *buildmer* (v2.11) begins with the identification of the “maximal model” such that the model fit converges, and then it performs stepwise backward elimination [37]. The method yielded the model containing the following variables: presence of subretinal fluid in the macula (*p* < 0.001), FAZ eccentricity (*p* < 0.001), no ischemic heart disease (*p* < 0.001), insulin (*p* < 0.001), past anti-VEGF (*p* < 0.001), integrity of ellipsoid zone in central 3 mm (*p* < 0.001), maximum FAZ distance corrected (*p* = 0.108), and pseudophakic (*p* = 0.930). The last two variables were discarded because neither had a statistically significant association with PNP. This final model yields perfect classification (AUC = 1.000; CI [1.000, 1.000]).

### 3.3. Predictors of Peripheral Non-Perfusion in DME—Summarized

**Figure 3:**
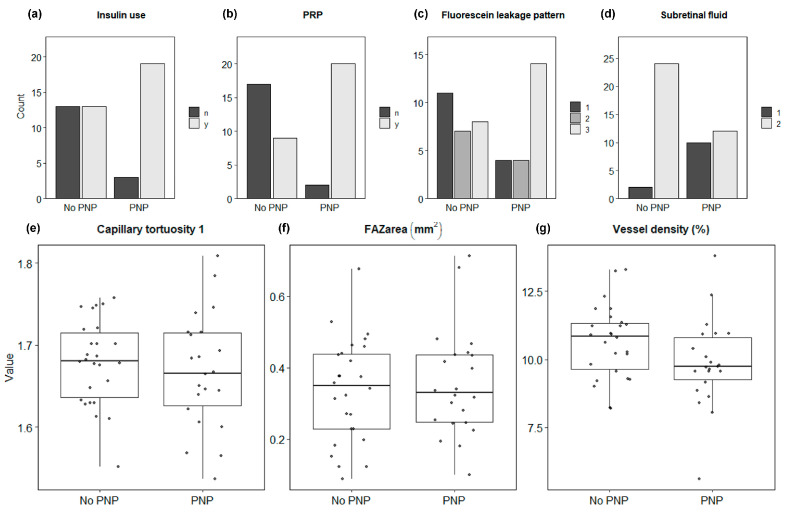
Graphical presentation of key observations in eyes with and without peripheral non-perfusion (PNP) in the setting of untreated diabetic macular edema. Categorical observations (**a**–**d**) from clinical and imaging data were found to be significantly associated with the presence of peripheral non-perfusion. Insulin use ((**a**); number of participants; y = yes; n = no; *p* = 0.013), pan-retinal photocoagulation (PRP) ((**b**); y = yes; n = no; *p* = 0.0003), subretinal fluid in the central macula ((**c**); 1 = present; 2 = absent; *p* = 0.0058), and fluorescein leakage pattern ((**d**); 1 = focal, >67% leakage from microaneurysms; 2 = intermediate; 3 = diffuse, <33% leakage from microaneurysms; *p* = 0.013). Key macula vascular metrics derived from optical coherence tomography angiography of the foveal avascular zone (FAZ) (**e**–**g**) ((**e**), FAZ area; (**f**) macula vessel density; (**g**) macula capillary tortuosity), fail to reach statistical significance (all *p* > 0.05). PNP group, n = 22; no PNP group, n = 26.

**Figure 4:**
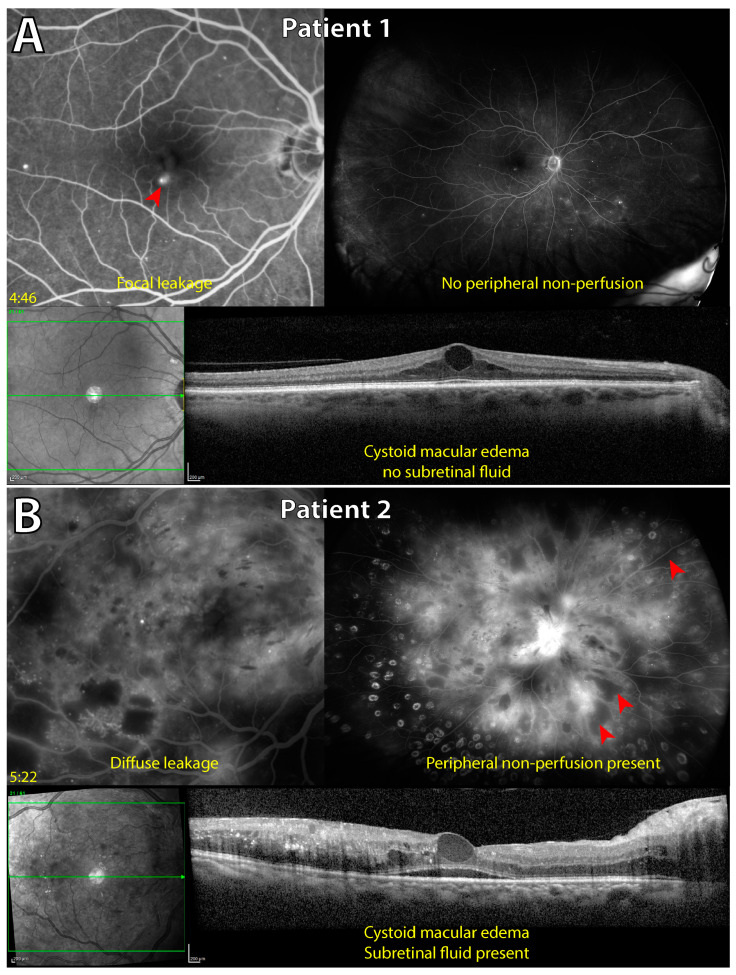
Association between a diffuse macular fluorescein leakage pattern and subretinal fluid with presence of peripheral non-perfusion. Multi-modal imaging of two separate eyes with diabetic macular edema (DME) from different patients. Late-phase fluorescein angiography in 30-degree and ultrawide field fields of view are shown, along with a structural optical coherence tomography (OCT) B-scan through the fovea. Patient 1 ((**A**); 64 years old) demonstrates a focal leakage pattern primarily from a single microaneurysm (red arrowhead) in the fovea with no peripheral non-perfusion present. Patient 2 ((**B**); 35 years old) manifests a diffuse fluorescein leakage pattern (<33% leakage from microaneurysms) with the presence of non-perfusion peripherally (red arrowheads). Subretinal fluid is present in the central macula on OCT only in patient 2 (**B**), in which peripheral non-perfusion is present. Both patients are type 2 diabetic, have a HbA1c of 6.4 and 10.7%, respectively, and only patient 2 (**B**) was prescribed insulin.

**Univariate analysis** using generalized linear mixed modeling comparing groups, PNP present versus PNP absent, was performed and yielded results as follows:

- Patient factors of insulin use (*p* < 0.001) and presence of PRP treatment (*p* < 0.001) were significantly different between groups (Figure 3). The data show insulin use and presence of PRP treatment to be more common in eyes with PNP.
- The pattern of fluorescein leakage, as seen on UWF FA, was significantly different between groups (*p* = 0.013) (Figure 3 and Figure 4). The data show diffuse leakage is more common in eyes with PNP.

**Multivariate analysis** using backward elimination to determine the best subset of variables that predict the presence of PNP found that the following subset yields perfect classification (AUC = 1.000; CI [1.000, 1.000]):

- Presence of subretinal fluid in central macula (*p* < 0.001) (Figure 3 and Figure 4).
- Disrupted ellipsoid zone in central macula (*p* < 0.001).
- Lower eccentricity of the FAZ (*p* < 0.001).
- Past anti-VEGF therapy (*p* < 0.001).
- Insulin use (*p* < 0.001) (Figure 3).
- Absence of ischemic heart disease diagnosis (*p* < 0.001).

### 3.4. Macular Vascular Metrics and Peripheral Non-Perfusion in DME

**Figure 5:**
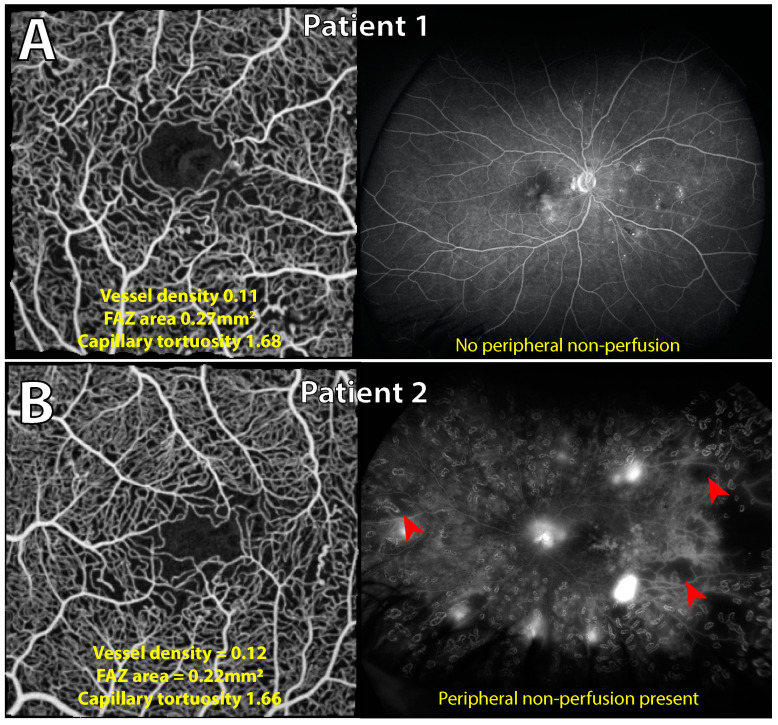
Central macula vascular metrics are not associated with the presence of peripheral non-perfusion in the setting of diabetic macular edema. Contemporaneous central and peripheral retinal vascular imaging in two eyes with diabetic macular edema from different patients is shown. Patient 1 ((**A**); 61 years old) and patient 2 ((**B**); 46 years old) demonstrate similar quantitative metrics of vessel density, foveal avascular zone (FAZ) area, and capillary tortuosity on 3 × 3 mm optical coherence tomography angiography (OCTA) scans. Qualitatively, there is no obvious disruption to the terminal capillary ring or gross perifoveal capillary loss in either OCTA image of the central macula. Despite this, on ultrawide field fluorescein angiography, patient 1 has no evidence of peripheral non-perfusion, whilst patient 2 has significant capillary non-perfusion peripherally (red arrowheads). Both patients are type 2 diabetic with comparable HbA1c values (7.9 and 8.3%, respectively) and normal renal function; only patient 2 was prescribed insulin.

None of the OCTA metrics investigated here, both quantitative and qualitative, were found to be significantly different between PNP present and absent groups in the univariate tests at α = 5% (Table 2 and Table 3) (Figure 3 and Figure 5). Observationally, of all the eyes with a “normal” or preserved perfusion density (assuming a “normal” perfusion density measure of >0.45 based on median results from large population-based studies [39,40]), there were only three (15%) eyes with PNP and 17 (85%) without PNP. Conversely, out of all eyes with a “reduced” macular perfusion density (<0.45), there were 19 (61%) eyes with PNP and a large minority of eyes without PNP, at 12 (38.7%). When applying a similar approach for FAZ area with <0.3 mm^2^ considered the “preserved” FAZ area (a common median for control eyes [40,41]), eight (42.1%) had PNP and for eyes with an “enlarged” FAZ area (>0.3 mm^2^), 14 (45.2%) had PNP.

## 4. Discussion

DR is one of the most significant causes of vision loss amongst working-age individuals [1]. Substantial vision loss in DR occurs primarily due to complications of the macula such as DME, diabetic macular ischemia, and neovascular complications including retinal detachments or vitreous hemorrhages. Vascular changes to the peripheral retina such as PNP, where there is an observable drop out of peripheral capillary beds, are associated with the natural course of macular disease in DR [42,43,44], especially in relation to DME, proliferative disease, and the progression of non-proliferative DR [42,45,46]. One study reported treatment-naive eyes with retinal ischemia had 3.75 times increased odds of having DME [42]. A 4-year prospective study reported a hazard ratio of 1.72 for the presence of peripheral lesions on UWF FA in eyes that manifested the progression of NPDR [47], and the degree of peripheral ischemia has been associated with DR severity [43,44]. Further studies found that the extent of PNP is associated with the risk of proliferative disease [45] and severity of DME [46]. Additionally, peripheral ischemic changes are known to predict anatomic treatment outcomes of anti-VEGF agents for DME [5], which are currently the preferred first-line treatment option. Knowledge of a patient’s peripheral retinal circulation is therefore valuable in the management of DR and its vision-threatening complications.

Routinely examining the peripheral microvasculature is challenging without FA and the invasive administration of intravenous contrast dyes. Wide-field OCTA is non-invasive and enables views not obscured by leakage but requires excellent fixation, is prone to artifacts, and has a relatively limited field of view when compared to UWF FA [48,49]. Current wide-field OCTA devices have a field of view from 50 to 100 degrees, whilst ultrawide field imaging devices may capture upward of 220 degrees [49]. A limited number of investigations have therefore explored the association of central macular vascular changes which are more easily visualized, with the presence and degree of peripheral retinal disease, specifically PNP. The COPRA study retrospectively analyzed 82 eyes with varying DR severities, including those with DME, and analyzed them collectively [26]. The authors found the PNP degree and FAZ metrics of macular ischemia to be associated, whilst macular vessel density was not significantly different. Interestingly, the association was only significant between the most severe tertile of PNP and the second tertile, whilst the least severe tertile did not manifest significant differences in macular metrics to even the most severe tertile [26]. In other words, the paper suggests a non-linear relationship of PNP severity and FAZ metrics of macular ischemia [26]. One cross-sectional analysis from 47 eyes both with and without DME found only a moderate correlation (*r* = 0.49) between the peripheral ischemic index and FAZ area, as derived from FA [27]. A small prospective pilot study of 22 eyes similarly found a moderate correlation between the peripheral ischemic index and FAZ area using OCTA (*r* = 0.60) [28]. Another prospective study, which excluded eyes with DME, found a moderate correlation with a novel macular non-perfusion metric called geometric perfusion deficit and PNP (*r* = 0.48) [29]. Contrary to these findings, a retrospective analysis (which explicitly excluded clinically significant DME) found no association between central ischemic metrics derived from OCTA (FAZ area and vessel density) and the peripheral ischemic index [30].

These papers have examined the same question under varying DR subtypes, some with both DME and non-DME eyes analyzed collectively, whilst the two studies that excluded clinically significant macular edema found conflicting results. Eyes with DR that develop DME may be seen as a particular subtype or phenotype of diabetic retina disease, as is evidenced by the fact that DME may occur at any stage throughout DR severity, is multifaceted in its pathogenesis, and manifests heterogenous treatment responses to anti-VEGF therapy [4,5,50]. Exploring relationships between DME and PNP may improve our understanding of the pathogenesis of DR complications and unravel novel associations with PNP using central biomarkers of DME and macular ischemia. The present study investigates the predictive power of qualitative and quantitative multi-modal contemporaneous imaging biomarkers in a prospective manner, specifically in the setting of eyes with DME, prior to the administration of anti-VEGF, steroid, or other intravitreal therapy [51]. Importantly, clinico-demographic factors, which may be superior to imaging biomarkers, were also investigated for their efficacy to predict the presence of PNP.

This study examined a wide range of biomarkers in the setting of DME, including macular vascular metrics, as assessed quantitatively and qualitatively with OCTA. In univariate analysis, none of the central vascular factors, including FAZ metrics, vessel density, or tortuosity, were statistically different between eyes with and without PNP. Only in multivariate analysis, where predictive variables are considered together and within-subject correlations are accounted for, did a central vascular metric, namely FAZ eccentricity, demonstrate significant predictive power for PNP (as part of a final model with six variables). In this model, lower FAZ eccentricity was associated with PNP; factors that can lead to eccentric distortion of the FAZ such as the mass effect of foveal cystoid DME [52] or mechanical traction [53] may therefore be driven by mechanisms independent of ischemic cytokines released in the setting of PNP. More commonly investigated vascular metrics, such as macular vessel density and FAZ area, showed no significant difference or significant predictive power in either analysis. Observationally, there appears to be a higher proportion of eyes (61%) with PNP in the “reduced” macular vessel density group, but there remains a large minority of eyes with no PNP (38.7%) despite a reduction in macular density. Furthermore, eyes with PNP were equally distributed between groups with “preserved” (42.1%) and “enlarged” FAZ areas (45.2%). Nevertheless, under more rigorous statistical examination using generalized linear mixed modeling, none of these observation differences are statistically significant.

Vascular tortuosity has seldom been investigated in relationship to PNP in DME. Factors that are thought to influence vessel tortuosity include those that are associated with PNP such as retinal hypoxia and altered vascular flow or resistance [31,32,33]. Histological analysis of diabetic human and rodent retina additionally reveals the degradation of key vessel components including pericytes and smooth muscle and endothelial intracellular cytoskeletal filaments [54,55,56,57,58,59]. These cellular structures help preserve vessel structure and integrity, the breakdown of which may increase susceptibility to tortuous changes in the setting of altered flow dynamics due to PNP. Presently, we find that the tortuosity of arterioles, capillaries, and venules in the central macula were not associated with the presence of PNP. The downstream effects of PNP on retinal hypoxia and macular flow dynamics may be insufficient to induce tortuous changes or may be adequately compensated for with the presence of intraretinal microvascular abnormalities (IRMAs) acting as shunt vessels [60].

In this particular subtype of diabetic retina disease (eyes exhibiting DME), vascular changes in the central fovea area appear unable to predict the presence of PNP. This is likely a reflection of the significant differences in the pathophysiology of capillary non-perfusion and DME and between neuronal and vascular anatomy and the physiology of central and peripheral retinas. The vascular layup of the macula contains four plexuses and is markedly different than the peripheral retina, which at the far periphery thins down to only a single plexus [61,62]. This difference alone could result in markedly divergent outcomes of ischemic metrics centrally and peripherally, even if the pathogenesis of vessel occlusion centrally and peripherally is shared. The few studies in this field discussed earlier provide conflicting evidence for correlations between central macular vessel changes and PNP [26,27,28,29,30]. Many of these reports support a mild–moderate correlation and postulate a common pathogenic link between central and peripheral ischemia [26,27,28,29,30]; none have specifically investigated this exclusively in eyes with DME, in which we present alternate findings. DME arises in specific eyes due to a combination of factors not entirely understood, including the breakdown of the inner and outer blood retinal barrier (BRB) affected by inflammatory and angiogenic chemokines and cytokines and Muller glia dysfunction resulting in a fluid inflow/outflow imbalance [2,63]. Retinal neurons, glia, and vessels are interrelated and operate as a neurovascular unit [64]. The same mechanisms that cause the localization of edematous changes to the macula, such as dysfunctional macular Muller cells, may therefore be exerting effects on the macular microvasculature which are not apparent in the peripheral retina. The significance is that central and peripheral ischemia may develop independently of each other in DME-prone eyes due to the unique environment in the macula. Accordingly, equivalent proportions of PNP are seen in eyes with preserved (42.1%) and enlarged FAZ areas (45.2%).

Other notable findings in the present report include the significant association of structural OCT biomarkers with the presence of PNP in the eyes with DME. Findings of subretinal fluid and disruption of the photoreceptor ellipsoid zone in the central macula were two observations of the multivariate subset of six variables that predicted PNP. Both of these changes occur toward the outer retina and may be sequelae of the outer BRB (retinal pigment epithelium) compromise, leading to fluid accumulation below the retina and an outer retinal environment in which the highly metabolic inner photoreceptor segment, rich with mitochondria and free radicals, is unable to survive. The association of these findings with PNP may be explained by the greater hypoxic load caused by peripheral ischemia, leading to the promotion of vasogenic factors like VEGF, which affect the permeability and barrier function of the retinal pigment epithelium [65]. Furthermore, univariate analysis suggested eyes with PNP more commonly manifested a diffuse pattern of fluorescein leakage on FA in the macula. Since our investigation specifically took place prior to administration of intravitreal anti-VEGF, this finding (and those of the outer retina) is likely explained by the greater permeability of the inner (and outer) BRB caused by vasogenic molecules like VEGF. Inflammatory processes that contribute to capillary non-perfusion through leukostasis, manifesting as PNP, may also be concurrently affecting inner and outer BRB function through inflammatory cytokines like interleukin-6, leading to DME and the findings of a diffuse leakage pattern, subretinal fluid, and ellipsoid zone disruption [66,67].

Our investigation included an analysis of patient and demographic factors beyond just imaging features. Insulin use, past anti-VEGF therapy, PRP, and no ischemic heart disease were four of the six factors in the multivariate predictive model. Insulin use was additionally more common in PNP present eyes in univariate analysis. This is consistent with the literature showing that the use of insulin in type 2 DM patients is associated with higher DR risk, severity, and DME [68,69]. Eyes with PNP had greater HbA1c levels (9.3%) than eyes without PNP (8.2%), though this difference was not significant. In this investigation, we only recruited those who had not had anti-VEGF, steroid, or laser therapy in the past six months. The predictive power of past anti-VEGF therapy for PNP may be attributed to the relationship PNP has with the severity of DR and the increased risk of DME development [42]. Those with past anti-VEGF therapies imply repeat episodes of DME, which PNP may be a risk factor for [42]. Interestingly, the absence of ischemic heart disease was one variable of the best subset to predict PNP. This may highlight the divergent pathophysiologic pathways of microvascular and macrovascular disease. Ischemic heart disease is most often due to an occlusive atherosclerotic process in the coronary arteries [70]. Microvascular disease at the level of retinal capillaries shows no evidence of atherosclerotic plaque development for many reasons, including the absence of vascular smooth muscle cells, absence of tunica media, and intima and lower shear stresses [70]. Capillaries are instead susceptible to endothelial dysfunction and leukostasis, leading to non-perfusion areas [67,71]. It is possible that medications and lifestyle interventions prescribed after a myocardial ischemic event may be beneficial to retinal vasculature despite the presence of overt macrovascular disease.

A major strength of our study is the prospective use of state-of-the-art contemporaneous multi-modal imaging used to create an exhaustive list of high-quality observations in order to determine the best predictors of PNP in the setting of DME. Limitations of this study include the relatively small sample size and observations compared to the number of variables investigated. This raises challenges or complications (collectively known as the curse of dimensionality) including computational problems trying to fit a complex model to the data and overfitting. This was the rationale for performing stepwise variable selection to determine the most discriminatory subset of variables. Nevertheless, the dimensionality of the final model is still large (six variables), so its classification accuracy for new unseen data remains an open question. A stratified analysis of different DM types was not included due to the sample size limitations presented by performing such a distinction. Data imputation was required on a small number of cases to maximize the number of observations available. Depth-resolved information from OCTA analysis in different vascular plexuses would be optimal but due to layer segmentation challenges in the setting of DME, we chose to only analyze the entire macular vascular slab. The data provided here are primarily relevant for the subset of diabetic retina disease patients susceptible to DME and no stratification of PNP severity was performed, because our primary question was to determine overall PNP status in this cohort. Further large-scale, longitudinal prospective studies may uncover significant predictive power in certain variables that were not apparent in this study. These larger studies are not easily feasible due to the challenges of performing contemporaneous multi-modal retinal imaging and recording of the exhaustive clinical observations in a large number of participants.

## 5. Conclusions

It is clear that central and peripheral retinal changes share a complex relationship in the unique retinal environment of DME. From the present study, it is apparent that central and peripheral vascular metrics are not always correlated. Importantly, this relationship may differ in non-DME patients, which are likely characterized by different disease pathways. Our findings do not support the concept that it is adequate to judge the status of the peripheral circulation using macular anatomic features alone in DME. Therefore, UWF FA and wide-field OCTA serve an important role by enabling the direct assessment of the peripheral microcirculation in these patients.

## Figures and Tables

**Table 1:** Findings of clinico-demographic variables grouped by the presence and absence of peripheral non-perfusion in eyes with diabetic macular edema. Numerical values are presented as mean ± standard deviation. *p*-values for Fisher’s exact test or independent samples *t*-test are shown. For the cases where the *p*-value is significant at α = 10%, the *p*-value obtained using the fitted univariate (generalized) linear mixed effects model (see the text for details) is shown in brackets.

Variable	PNP Present	PNP Absent	*p*-Value
Demographic and Clinical Details			
Sample size	22	26	-
Age (years)	52 ± 15	59 ± 12	0.075 * (0.145)
Sex (# male)	12 (50%)	20 (80%)	0.232
Visual acuity (ETDRS letters)	69.1 ± 16.1	76.5 ± 11.3	0.11
DM type (# type 2)	14 (63.6%)	22 (84.6%)	0.36
Duration of DM (years)	22 ± 7.5	18.8 ± 9.4	0.20
HbA1c (%)	9.3 ± 2.2	8.2 ± 2.0	0.13
Insulin use	18 (81.8%)	14 (53.8%)	0.013 ** (0.0001 **)
Smoking	6 (27.2%)	6 (23.1%)	0.73
eGFR (mL/min/1.73 m^2^)	78.5 ± 21.2	67.7 ± 22.9	0.12
Creatinine (µmol/L)	83.2 ± 42.3	117.3 ± 110.5	0.16
LDL (mmol/L)	2.8 ± 0.8	2.6 ± 1.2	0.58
HDL (mmol/L)	1.3 ± 0.4	1.2 ± 0.4	0.5
Lipid-lowering therapy use	9 (40.9%)	18 (69.2%)	0.24
Hypertension diagnosis	15 (70%)	20 (80%)	1
Ischemic heart disease	2 (9.1%)	7 (26.9%)	0.26
Stroke	0 (0%)	3 (11.5%)	0.49
PRP (pan-retinal photocoagulation)	20 (90%)	10 (40%)	<0.0001 (0.0003 **)
Pseudophakic	5 (22.7%)	7 (26.9%)	1
Past intravitreal anti-VEGF	10 (45.4%)	9 (34.6%)	0.37
Past intravitreal steroids	1 (4.5%)	1 (3.8%)	1
Previous vitrectomy	1 (4.5%)	0 (0%)	0.46

* significance at α = 10%; ** significance at α = 5%. DM = diabetes mellitus; PRP = pan-retinal photocoagulation; VEGF = vascular endothelial growth factor; PNP = peripheral non-perfusion.

**Table 2:** Findings of graded imaging features grouped by the presence and absence of peripheral non-perfusion in eyes with diabetic macular edema. Data are presented as the number of participants per group. *p*-values for Fisher’s exact test or independent samples *t*-test are shown. For the cases where the *p*-value is significant at α = 10%, the *p*-value obtained using the fitted univariate (generalized) linear mixed effects model (see the text for details) is shown in brackets.

Variable	PNP Present	PNP Absent	*p*-Value
Imaging Features Graded by Reviewers			
Presence of hard exudates in macula	11 (50%)	14 (53.8%)	0.78
Microaneurysms	0 (0%) none	0 (0%) none	0.007 ** (singular model)
	12 (54.5%) <10	5 (19.2%) <10	
	10 (45.5%) 10 or greater	23 (88.5%) 10 or greater	
Fluorescein leakage pattern in the macula	3 (13.6%) focal	12 (46.2%) focal	0.067 * (0.013 **)
	4 (18.2%) intermediate	7 (26.9%) intermediate	
	15 (68.2%) diffuse	9 (34.6%) diffuse	
Subretinal fluid presence	10 (45.5%)	2 (7.7%)	0.0058 ** (0.618)
Cystoid changes in inner nuclear layer	14 (63.6%)	19 (73.1%)	0.54
Cystoid changes in outer nuclear layer	19 (86.4%)	26 (100%)	0.089 *
Intact ellipsoid zone	19 (86.4%)	26 (100%)	0.649
Presence of DRiL	5 (22.7%)	9 (34.6%)	0.52
Hyper-reflective foci count	6 (27.2%) 0	3 (11.5%) 0	0.86
	6 (27.2%) <10	11 (42.3%) <10	
	4 (18.2%) 10–20	5 (19.2%) 10–20	
	6 (27.3%) >20	7 (26.9%) >20	
Intact terminal foveal capillary ring	4 (18.2%)	10 (38.5%)	0.52
Perifoveal capillary loss	19 (86.4%)	25 (96.2%)	0.39

* significance at α = 10%; ** significance at α = 5%. DRiL = disorganization of retinal inner layers; PNP = peripheral non-perfusion.

**Table 3:** Findings of quantitative imaging variables grouped by the presence and absence of peripheral non-perfusion in eyes with diabetic macular edema. Numerical values are presented as mean ± standard deviation. *p*-values for Fisher’s exact test or independent samples *t*-test are shown. For the cases where the *p*-value is significant at α = 10%, the *p*-value obtained using the fitted univariate (generalized) linear mixed effects model (see the text for details) is shown in brackets.

Variable	PNP Present	PNP Absent	*p*-Value
Quantitative Imaging Features			
Axial length (mm)	23.4 ± 1.02	23.7 ± 0.94	0.39
Central retinal thickness (µm)	403.1 ± 129.8 (1 mm)	375.4 ± 120.4 (1 mm)	0.55
	383.5 ± 144 (3 mm)	388.2 ± 88.7 (3 mm)	0.29
	358.2 ± 230(6 mm)	366.4 ± 126 (6 mm)	0.28
Vessel tortuosity method 1	1.66 ± 0.11 (arterioles)	1.67 ± 0.06 (arterioles)	0.62
	1.66 ± 0.11 (venules)	1.67 ± 0.06 (venules)	0.58
	1.67 ± 0.07 (capillaries)	1.68 ± 0.05 (capillaries)	0.54 (0.55)
Vessel tortuosity method 2	1.06 ± 0.12 (arterioles)	1.06 ± 0.11 (arterioles)	0.84
	1.05 ± 0.14 (venules)	1.06 ± 0.12 (venules)	0.95
	1.04 ± 0.15 (capillaries)	1.025 ± 0.07 (capillaries)	0.62 (singular model)
Macular perfusion density	0.41 ± 0.05	0.45 ± 0.06	0.053 * (0.145)
Macular vessel density	0.097 ± 0.014	0.106 ± 0.014	0.077 * (0.152)
Average macular vessel diameter (mm)	0.024 ± 0.002	0.023 ± 0.002	0.95
Macular fractal dimension	1.88 ± 0.01	1.89 ± 0.01	0.2
Minimum FAZ distance (mm)	0.49 ± 0.14	0.45 ± 0.13	0.19
Maximum FAZ distance (mm)	0.82 ± 0.21	0.81 ± 0.22	0.54
FAZ area (mm^2^)	0.36 ± 0.15	0.35 ± 0.16	0.65 (0.57)
FAZ eccentricity	0.65 ± 0.14	0.67 ± 0.12	0.3
FAZ axis ratio	1.73 ± 0.32	1.86 ± 0.42	0.23
FAZ perimeter (mm)	3.37 ± 1.17	3.43 ± 1.39	0.75
FAZ acircularity index	1.61 ± 0.29	1.67 ± 0.45	0.33

* significance at α = 10%. FAZ = foveal avascular zone; PNP = peripheral non-perfusion.

## Data Availability

The data presented in this study are available upon request from the corresponding author.

## Supplementary Materials

The following supporting information can be downloaded at: https://www.mdpi.com/article/10.3390/jcm14010052/s1, Supplementary Table S1: Source and unit/categorical definitions of observations; Supplementary Figure S1: Graphical representation of all numerical observations; Supplementary Figure S2: Graphical representation of all categorical observations; Supplementary Figure S3: Paired plots data of OCTA quantitative metrics.
