# Supplementary material for: Predictors of Peripheral Retinal Non-Perfusion in Clinically Significant Diabetic Macular Edema
Source: J Clin Med. 2024 Dec 26;14(1):52. doi: 10.3390/jcm14010052 (PMC11722121; doi:10.3390/jcm14010052)
Supplement: Supplementary file 1 [file jcm-14-00052-s001.zip › jcm-3354984-supplementary.docx]

**Supplementary Table S1.** Source and unit/categorical definitions of observations.

| Variable (shortened name) | Units / categories | Source |
| --- | --- | --- |
| Demographic and clinical details | | |
| Patient ID | Categorical | - |
| Age | Years | Clinical details/demographics |
| Sex | Categorical (M/F) | Clinical details/demographics |
| Visual acuity | ETDRS | Clinical details/demographics |
| DM type | Categorical (1/2) | Clinical details/demographics |
| Duration of DM | Years | Clinical details/demographics |
| HbA1c | % | Clinical details/demographics |
| Insulin use | Categorical (Y/N) | Clinical details/demographics |
| Smoking | Categorical (Y/N) | Clinical details/demographics |
| eGFR | mL/min/1.73m2 | Clinical details/demographics |
| Creatinine | µmol/L | Clinical details/demographics |
| LDL | mmol/L | Clinical details/demographics |
| HDL | mmol/L | Clinical details/demographics |
| Lipid-lowering therapy use (lipid therapy) | Categorical (Y/N) | Clinical details/demographics |
| Hypertension | Categorical (Y/N) | Clinical details/demographics |
| Ischemic heart disease | Categorical (Y/N) | Clinical details/demographics |
| Stroke | Categorical (Y/N) | Clinical details/demographics |
| PRP | Categorical (Y/N) | Clinical details/demographics |
| Pseudophakic | Categorical (Y/N) | Clinical details/demographics |
| Past intravitreal anti-VEGF (past anti-VEGF) | Categorical (Y/N) | Clinical details/demographics |
| Past intravitreal steroids | Categorical (Y/N) | Clinical details/demographics |
| Previous vitrectomy | Categorical (Y/N) | Clinical details/demographics |
| Imaging features graded by reviewer | | |
| Presence of exudates | Categorical (Y/N) | Fundus photography, OCT |
| Microaneurysms | Categorical (none, <10 or 10 or greater) | Fundus photography, OCT |
| Fluorescein leakage pattern in the macula (fluorescein leakage pattern) | Categorical (focal >66% leakage from microaneurysms, intermediate 33-66% leakage from microaneurysms or diffuse <33% leakage from microaneurysms) | FA |
| Peripheral non-perfusion | Categorical (Y/N) | UWF FA |
| Subretinal fluid | Categorical (Y/N) | OCT |
| Cystoid changes in INL | Categorical (Y/N) | OCT |
| Cystoid changes in ONL | Categorical (Y/N) | OCT |
| Intact ellipsoid zone | Categorical (Y/N) | OCT |
| Presence of DRiL | Categorical (Y/N) | OCT |
| Intraretinal HRF (HRF) | Categorical (Y/N) | OCT |
| Intact terminal foveal capillary ring (intact FAZ capillaries) | Categorical (Y/N) | OCTA |
| Perifoveal capillary loss | Categorical (Y/N) | OCTA |
| Quantitative Imaging Features | | |
| Axial length | mm | IOL master |
| Central retinal thickness (1mm CRT, 3mm CRT, 6mm CRT) | µm | OCT |
| Vessel tortuosity method 1 (arterioles tortuosity 1, venules tortuosity 1, capillary tortuosity 1) | Numerical | OCTA |
| Vessel tortuosity method 2 (arterioles tortuosity 2, venules tortuosity 2, capillary tortuosity 2) | Numerical | OCTA |
| Average vessel diameter | Numerical | OCTA |
| Perfusion density | Numerical | OCTA |
| Vessel density | Numerical | OCTA |
| Fractal dimension | Numerical | OCTA |
| Minimum FAZ distance | mm | OCTA |
| Maximum FAZ distance | mm | OCTA |
| FAZ area | mm^2^ | OCTA |
| FAZ eccentricity | Numerical | OCTA |
| FAZ axis ratio | Numerical | OCTA |
| FAZ perimeter | mm | OCTA |
| FAZ acircularity index | Numerical | OCTA |


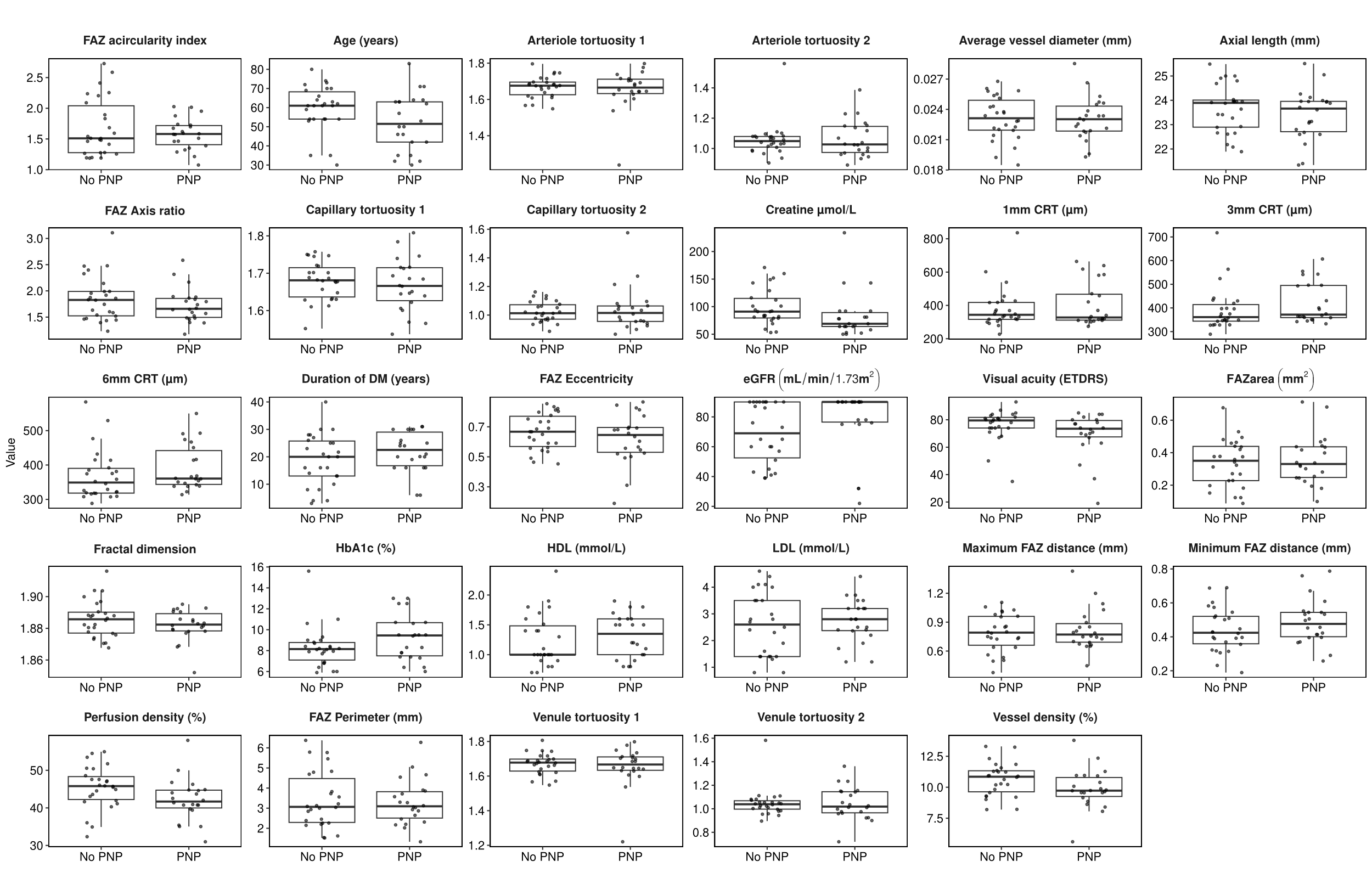


**Supplementary Figure S1.** Graphical representation of all numerical observations recorded from demographic, clinical, and imaging data. Grouped into either peripheral non-perfusion (PNP) present or PNP absent groups.


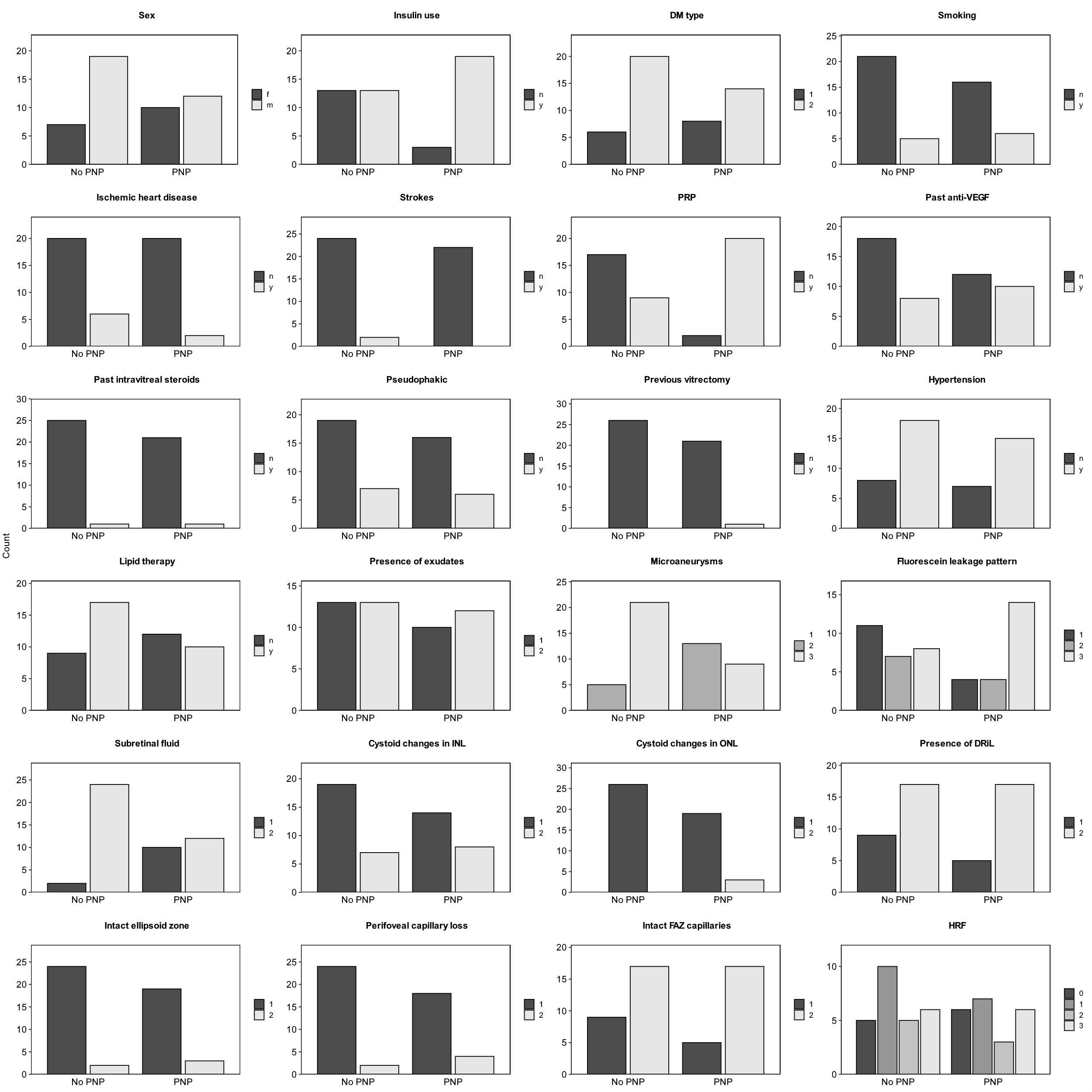


**Supplementary Figure S2.** Graphical representation of all categorical observations recorded from demographic, clinical, and macular imaging data. Grouped into either peripheral non-perfusion (PNP) present or PNP absent groups. Data are presented as (count) number of participants in the category of each group. Exudates: 1 = present; 2 = absent. Microaneurysm count in the macula: 1 = absent; 2 = <10; 3 = >10. Fluorescein leakage pattern in the macula: 1 = focal; 2 = intermediate; 3 = diffuse. Subretinal fluid in the macula: 1 = present; 2 = absent. Cystoid changes in INL: 1 = present; 2 = absent. Cystoid changes in ONL: 1 = present; 2 = absent. Disruption of retinal inner layers (DRiL): 1 = present; 2 = absent. Integrity of ellipsoid zone: 1= intact; 2 = disrupted. Perifoveal capillary loss: 1 = present; 2 = absent. Intact foveal avascular zone (FAZ) terminal capillaries: 1 = intact; 2 = disrupted. Hyperreflective foci (HRF) count: 0 = none; 1 = <10; 2 = 10-20; 3 = >20.

**Supplementary Figure S3.** Paired plots of optical coherence tomography angiography quantitative metrics. Strong correlations are demonstrated between venule and arteriole tortuosity values and perfusion density and vessel density.
